# Editorial: Non-invasive Brain Stimulation in the Study and Modulation of Metaplasticity in Neurological Disorders

**DOI:** 10.3389/fneur.2021.721906

**Published:** 2021-06-30

**Authors:** Mariagiovanna Cantone, Giuseppe Lanza, Federico Ranieri, George M. Opie, Carmen Terranova

**Affiliations:** ^1^Department of Neurology, Sant'Elia Hospital, ASP Caltanissetta, Caltanissetta, Italy; ^2^Department of Surgery and Medical-Surgical Specialties, University of Catania, Catania, Italy; ^3^Department of Neurology IC, Oasi Research Institute–IRCCS, Troina, Italy; ^4^Unit of Neurology, Department of Neuroscience, Biomedicine and Movement Sciences, University of Verona, Verona, Italy; ^5^Discipline of Physiology, Adelaide Medical School, The University of Adelaide, Adelaide, SA, Australia; ^6^Department of Clinical and Experimental Medicine, University of Messina, Messina, Italy

**Keywords:** transcrancial magnetic stimulation, direct current stimulation, neuroplasicity, stroke rehabilitation, depression treatment

This Research Topic, which consists of 8 articles by a total of >40 authors, addresses different aspects of metaplasticity in acquired neurological and psychiatric disorders. Metaplasticity refers to the activity-dependent modulation of synaptic plasticity. This pivotal determinant of learning, memory, and other functions represents a higher order of synaptic plasticity that acts on the threshold for modifying synaptic strength (1). However, our understanding of the cellular and molecular mechanisms underlying distinct forms of synaptic plasticity, including metaplasticity, remains limited. Moreover, impaired synaptic plasticity, the so-called “maladaptive plasticity,” has been associated with the pathogenesis and trajectory of several brain diseases, including contributions to the dysfunctional remodeling of underlying neural networks (2–5).

Given its role in regulating synaptic plasticity, alterations to metaplastic mechanisms are likely to represent an important element of many neurological disorders. Until relatively recently, though, investigation of these processes was limited to invasive techniques in animal models. However, the development of non-invasive brain stimulation techniques (NIBS) has meant that it is now possible to induce and modulate metaplasticity in human subjects. Excitingly, there is a rapidly growing constellation of novel interventions that have been developed using NIBS, many of which are showing promise as therapeutic tools for treating neurological and neuropsychiatric disorders, despite our still limited understanding of the contribution made by metaplasticity. In support of this, the study by Thomson and Sack reviewed studies utilizing transcranial magnetic stimulation (TMS, a form of NIBS involving magnetic pulses applied over the scalp) to study and modulate metaplasticity, with specific interest in clinical applications. In particular, they focused on the use of repetitive TMS (rTMS) with intermittent theta burst stimulation (iTBS) and continuous TBS, as these are two of the most known and applied stimulation paradigms within research and clinical settings. After reviewing the relevant literature, the authors concluded that there is indeed a great potential to develop metaplasticity-based treatments to induce or restore a desired level of synaptic plasticity. They further identified accelerated iTBS at longer intervals (60 min) as being of particular interest, as it seems to maximize metaplasticity effects and clinical outcomes.

While TMS was the original NIBS technique to be used for the investigation of metaplasticity, the more recently developed transcranial direct (tDCS) and alternating (tACS) current stimulation, both of which involve low intensity electrical stimulation to the scalp, have also become widely applied within this field. In the study by Korai et al., the neurobiological mechanisms underlying the after-effects of tDCS and tACS was therefore reviewed. The authors discuss that, in contrast to TMS, these forms of NIBS do not produce action potentials in affected tissues. Instead, they modulate membrane potential within a sub-threshold range, and this leads to consequent changes in synaptic transmission. The role of meta-plasticity in mediating these effects is further discussed by the authors. In particular, the way in which synaptic efficacy is effectively modulated only when concurrent neuronal discharge take place (6, 7). This opens new insights on rehabilitation protocols based on concomitant NIBS and training-induced neuronal activation.

Although applied broadly across many clinical domains, there has been a preponderance of NIBS-based research in the area of stroke. In particular, the development of interventions able to promote functionally beneficial patterns of brain activity in stroke patients has been common, and this approach likely involves metaplastic mechanisms. In an alternative take on this goal, the study by Hamaguchi et al. instead aimed to identify if it is possible to predict participants that will benefit from a combination of NIBS and occupational therapy (OT) (i.e., “responders”) based on pre-treatment functional scores. In 1,254 patients with upper extremity post-stroke paralysis, the authors therefore assessed if the response to low frequency (i.e., 1 Hz) rTMS applied to the contralesional primary motor cortex (M1) immediately prior to OT could be predicted by pretreatment Fugl-Meyer Motor Assessment (FMA) scores of the upper limb. The intervention showed a facilitation of muscle movements by the rTMS-modulation of M1 excitability. Moreover, the probability of being non-responders was 59.2% when the initial FMA score was 48.9, whereas when the initial score was 38.8 the incidence of responders and hyper-responders was 45.5 and 16.0%, respectively. Notably, ~45% of the patients with FMA scores from 30 to 40 before treatment improved, and even >25% of those with more severe initial values. Overall, these results suggest that pretreatment assessment can estimate the possibility of a patient's recovery in the chronic phase, with relevant implications for therapists and patient's compliance and cooperation.

Using a slightly different approach that nonetheless highlights the utility of combining NIBS with functional interventions in stroke patients, the study by Zhong et al. tested how the site of stimulation influences recovery from dysphagia in subacute stroke patients. Specifically, the benefit of 5 Hz-rTMS combined with standard sensory-motor rehabilitation of dysphagia was compared when applying stimulation to the M1 and cerebellum of both affected and unaffected hemispheres. They reported that, relative to a non-stimulated control group, 2-weeks of combined stimulation and training resulted in improved recovery, and this was consistent across all sites of stimulation. This implies that rTMS may have stimulated the training-induced plasticity involved in swallowing control, possibly by acting on different circuits, although the specific pathomechanisms need to be clarified.

While a large amount of the literature utilizing NIBS in stroke has been focused on improving motor symptoms, the interesting study by Fray et al. instead evaluated the use of intense rTMS to treat post-stroke depression (PSD). In six subacute stroke patients, high-frequency (20 Hz) rTMS was applied over the left dorsolateral prefrontal cortex (DLPFC) during five sessions per day and over 4 consecutive days (20 sessions in total). At the end of the procedure and after 3 months, scores of depression significantly decreased, without any procedure-related adverse event. The authors concluded that, despite the small sample size of this pilot study, intense rTMS may be a safe and effective alternative or adjunctive therapy for PSD patients.

In further support of the cognitive benefits that are achievable when applying NIBS in the clinic, the elegant study by Sumiyoshi et al. determined whether tDCS improves semantic memory in schizophrenia patients, assessed using text-mining analyses of category fluency data. Indeed, semantic memory deficits have been previously reported in schizophrenia and associated with negative symptoms and quality of life. In 28 schizophrenia patients, cognitive assessment was carried out at baseline and 1 month after tDCS, which was performed twice per day for five consecutive days, with the anode electrode over the left DLPFC and cathode electrode over the right supraorbital area. After multi-session tDCS, the authors observed a normalization of semantic associations. The left prefrontal region is assumed to be related to the ability of tDCS to improve the organization of information and retrieval of clustered words, thus supporting the role of neuromodulation in improving cognitive functions in psychiatric disorders.

The third review within this edition also serves to demonstrate the cognitive benefits that can be derived from utilizing NIBS as an adjunctive therapy within a clinical population. Accordingly, the mini-review by Suarez-Garcia et al. sought to characterize the current evidence supporting the use of tDCS for treating cognitive impairment in Parkinson's disease (PD). A systematic review was used to identify 8 studies, the data from which was subsequently entered in to a meta-analysis. Although the results of this analysis were limited by the low number of studies and the heterogeneity of stimulation protocols and clinical features, they nonetheless identified strong benefits to executive functions in patients. In particular, anodal tDCS appears to improve problem solving and planning, verbal fluency, and cognitive flexibility.

Finally, an example of metaplastic modulation in clinical practice has been described in the case reported by Serrano-Castro et al. Despite an invasive neurostimulation approach, they opened the way to a customized neuroplastic-guided rehabilitation protocol, which allowed a previously inoperable tumor to be successfully removed and subsequently help treat the patient's refractory epilepsy.

In conclusion, this Research Topic includes a number of remarkable advances that further our understanding of the complex phenomena underlying metaplasticity, demonstrate how aberrant metaplasticity can contribute to pathophysiology, and show that modifying metaplasticity with NIBS can be an effective avenue for treating network disorders of the brain. Translationally, this will encourage future clinical and neurophysiological studies and open novel therapeutic perspectives in this fascinating topic.

## Author Contributions

MC, FR, and CT draft the manuscript. MC, GL, and CT conduct the analysis of data. GL and GO revise the manuscript critically for important intellectual content. All the authors approve the version of the manuscript to be published.

## Conflict of Interest

The authors declare that the research was conducted in the absence of any commercial or financial relationships that could be construed as a potential conflict of interest.
